# 5-Hy­droxy-7-phenyl-5-(prop-2-yn-1-yl)-5,6-dihydro-1-benzofuran-2(4*H*)-one monohydrate

**DOI:** 10.1107/S1600536810028400

**Published:** 2010-10-20

**Authors:** Laura Torre-Fernández, Marcos G. Suero, Santiago García-Granda

**Affiliations:** a Departamento de Química Física y Analítica, Facultad de Química, Universidad de Oviedo, C/ Julián Clavería, 8, 33006 Oviedo, Spain; b Departamento de Química Orgánica e Inorgánica, Facultad de Química, Universidad de Oviedo, C/ Julián Clavería, 8, 33006 Oviedo, Spain

## Abstract

In the title compound, C_17_H_14_O_3_·H_2_O, the six-membered ring, which adopts a half-chair conformation, makes a dihedral angle of 24.3 (2)° with the phenyl ring. In the crystal, the components are linked by O—H⋯O hydrogen bonds involving the water mol­ecule, and the hy­droxy and carbonyl groups of the organic compound. These inter­actions form a square-like supra­molecular synthon unit which propagates as chains parallel to the crystallographic *b* axis.  A C—H⋯O interaction also occurs.

## Related literature

For related literature about the cited reactions, see: Bassetti *et al.* (2005 ▶); Beck *et al.* (2001 ▶); Liu *et al.* (2006 ▶); Ma & Gu (2005 ▶); Rudler *et al.* (2004 ▶).
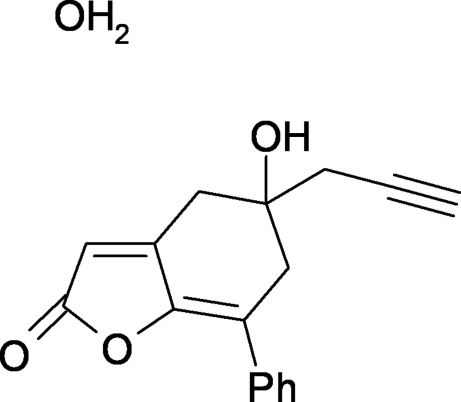


## Experimental

### 

#### Crystal data

C_17_H_14_O_3_·H_2_O
*M* *_r_* = 284.30
Monoclinic, 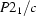

*a* = 9.1585 (2) Å
*b* = 9.2160 (3) Å
*c* = 17.4628 (5) Å
β = 91.145 (2)°
*V* = 1473.65 (7) Å^3^
*Z* = 4
Mo *K*α radiation
μ = 0.09 mm^−1^
*T* = 293 K
0.20 × 0.20 × 0.18 mm

#### Data collection

Nonius KappaCCD diffractometer
Absorption correction: refined from Δ*F* (*XABS2*; Parkin *et al.*, 1995 ▶) *T*_min_ = 0.982, *T*_max_ = 0.983
5821 measured reflections
3360 independent reflections
2251 reflections with *I* > 2σ(*I*)
*R*_int_ = 0.028

#### Refinement

*R*[*F* ^2^ > 2σ(*F* ^2^)] = 0.053
*wR*(*F* ^2^) = 0.191
*S* = 1.14
3360 reflections
195 parameters
3 restraints
H-atom parameters constrained
Δρ_max_ = 0.50 e Å^−3^
Δρ_min_ = −0.69 e Å^−3^

### 

Data collection: *COLLECT* (Nonius, 2000 ▶); cell refinement: *SCALEPACK* (Otwinowski & Minor, 1997 ▶); data reduction: *DENZO* (Otwinowski & Minor, 1997 ▶) and *SCALEPACK*; program(s) used to solve structure: *SIR2004* (Burla *et al.*, 2005 ▶); program(s) used to refine structure: *SHELXL97* (Sheldrick, 2008 ▶); molecular graphics: *ORTEP-3 for Windows* (Farrugia, 1997 ▶); software used to prepare material for publication: *WinGX* (Farrugia, 1999 ▶).

## Supplementary Material

Crystal structure: contains datablocks global, I. DOI: 10.1107/S1600536810028400/zq2046sup1.cif

Structure factors: contains datablocks I. DOI: 10.1107/S1600536810028400/zq2046Isup2.hkl

Additional supplementary materials:  crystallographic information; 3D view; checkCIF report

## Figures and Tables

**Table 1:** Hydrogen-bond geometry (Å, °)

*D*—H⋯*A*	*D*—H	H⋯*A*	*D*⋯*A*	*D*—H⋯*A*
O10—H10⋯O21	0.82	1.94	2.735 (2)	163
O21—H21*A*⋯O10^i^	1.00	1.74	2.728 (2)	169
O21—H21*B*⋯O14^ii^	1.00	1.75	2.754 (2)	176
C13—H13⋯O21^iii^	0.93	2.47	3.236 (3)	139

Symmetry codes: (i) 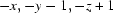; (ii) 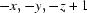; (iii) 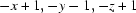.

**Fig. 1.**
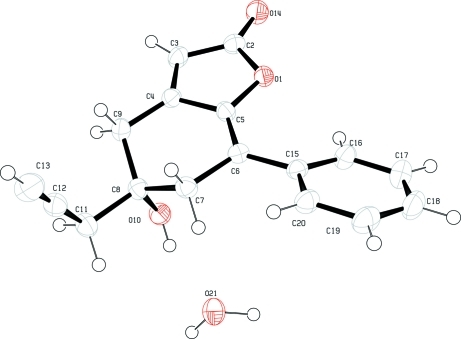
Perspective view of the title compound. Displacement ellipsoids are drawn at the 50% probability level.

## supplementary crystallographic information

### Comment

2-Butenolides are ubiquitous chemical moieties found in many natural products coming from plants, microorganisms and algae. A range of synthetic approaches to this class of compounds exists and includes among others, palladium-catalyzed cross-coupling reaction between allenoic acids and 2,3-allenols (Ma *et al.*, 2005), gold-catalyzed *Z*-enynol cyclization (Liu *et al.*, 2006) or ring-closing metathesis of methallyl acrylates (Bassetti *et al.*, 2005). However, only a few multicomponent methods have been reported that include a Passerini reaction (Beck *et al.*, 2001) and Fischer carbene complexes (Rudler *et al.*, 2004) among others. In this context, a new multicomponent method for the synthesis of bicyclic 2-butenolides through the coupling of imide lithium enolates, propargylic organometallics and Fischer carbene complexes will be soon published elsewhere.

The molecular structure of the title compound is shown in Fig. 1. The molecular packing is dominated by three main hydrogen bonds O10—H10···O21, O21—H21A···O10^i^ and O21—H21B···O14^ii^ involving the water molecule, and the hydroxyl and carbonyl groups of the compound. These interactions involving two compounds and two water molecules form a square-like supramolecular synthon unit which propagates as linear chains parallel to the crystallographic *b* axis.

### Experimental

n-Butyllithium (1.2 mmol, 1.6 *M* in hexane, 750 µ*L*) was added to a stirred solution of diisopropylamine (1.2 mmol, 172 µ*L*) in THF (2 ml) at 273 K. After stirring for 15 min at 273 K, the solution was cooled to 195 K and 3-acetyl-2-oxazolidinone (1.2 mmol, 155 mg) in THF (2 ml) was added dropwise over 5 min. The mixture was stirred at 195 K for a further 30 min period to complete the formation of the lithium enolate. Pentacarbonyl-(1-methoxy-1-phenylmethylene)chromium (1 mmol, 312 mg) in THF (20 ml) was added over the lithium enolate solution at 195 K and the resulting mixture was stirred for 15 min. After that, propargylmagnesium bromide (2.6 mmol, 0.5 *M* in Et_2_O, 5.2 ml) was added dropwise at 195 K. The mixture was stirred for 30 min at 195 K and then for 12 h at 218 K. Then it was allowed to reach 293 K slowly (8 h). The reaction was quenched with NH_4_Cl (20 ml, saturated aqueous solution), diluted with hexane/ethyl acetate, 10/1 (110 ml) and subjected to air oxidation under sunlight. After 24 h, the yellow suspension was filtered through Celite and extracted with diethyl ether (3 *x* 10 ml). The organic layers were combined, dried over anhydrous Na_2_SO_4_ and concentrated *in vacuo*. The crude product was purified by flash column chromatography on silica gel using mixtures of hexane/ethyl acetate (20/1 to 9/1 to 3/1 to 1/1) to yield the title compound (0.68 mmol, 181 mg, 68%) as a pure compound.

### Refinement

All non-H atoms were anisotropically refined. All H atoms were placed in geometrically idealized positions with C—H = 0.93 Å for the aromatic H atoms and for the acetylenic H atom, with C—H = 0.97 Å for the methylene H atoms, with O—H = 0.82 Å for the hydroxy H atom, and with O—H = 1.0 Å for the water H atoms. All of them were constrained to ride on their parent atoms with *U_iso_*(H) = 1.2**U_eq_*(C) and *U_iso_*(H) = 1.5**U_eq_*(O), except for the water H atoms which were isotropically refined.

### Crystal data

**Table:**

C_17_H_14_O_3_·H_2_O	*F*(000) = 600
*M**_r_* = 284.30	*D*_x_ = 1.281 Mg m^−^^3^
Monoclinic, *P*2_1_/*c*	Mo *K*α radiation, λ = 0.71073 Å
Hall symbol: -P 2ybc	Cell parameters from 3327 reflections
*a* = 9.1585 (2) Å	θ = 1.0–27.5°
*b* = 9.2160 (3) Å	µ = 0.09 mm^−^^1^
*c* = 17.4628 (5) Å	*T* = 293 K
β = 91.145 (2)°	Prismatic, colourless
*V* = 1473.65 (7) Å^3^	0.20 × 0.20 × 0.18 mm
*Z* = 4	

### Data collection

**Table:**

Nonius KappaCCD diffractometer	3360 independent reflections
Radiation source: Enraf–Nonius FR590	2251 reflections with *I* > 2σ(*I*)
horizonally mounted graphite crystal	*R*_int_ = 0.028
Detector resolution: 9 pixels mm^-1^	θ_max_ = 27.5°, θ_min_ = 2.2°
CCD rotation images, thick slices scans	*h* = −11→11
Absorption correction: part of the refinement model (Δ*F*) (*XABS2*; Parkin *et al.*, 1995)	*k* = −11→11
*T*_min_ = 0.982, *T*_max_ = 0.983	*l* = −22→22
5821 measured reflections	

### Refinement

**Table:**

Refinement on *F*^2^	Primary atom site location: structure-invariant direct methods
Least-squares matrix: full	Secondary atom site location: difference Fourier map
*R*[*F*^2^ > 2σ(*F*^2^)] = 0.053	Hydrogen site location: inferred from neighbouring sites
*wR*(*F*^2^) = 0.191	H-atom parameters constrained
*S* = 1.14	*w* = 1/[σ^2^(*F*_o_^2^) + (0.1074*P*)^2^] where *P* = (*F*_o_^2^ + 2*F*_c_^2^)/3
3360 reflections	(Δ/σ)_max_ < 0.001
195 parameters	Δρ_max_ = 0.50 e Å^−^^3^
3 restraints	Δρ_min_ = −0.69 e Å^−^^3^

### Special details

**Table:**

Geometry. All e.s.d.'s (except the e.s.d. in the dihedral angle between two l.s. planes) are estimated using the full covariance matrix. The cell e.s.d.'s are taken into account individually in the estimation of e.s.d.'s in distances, angles and torsion angles; correlations between e.s.d.'s in cell parameters are only used when they are defined by crystal symmetry. An approximate (isotropic) treatment of cell e.s.d.'s is used for estimating e.s.d.'s involving l.s. planes.
Refinement. Refinement of *F*^2^ against ALL reflections. The weighted *R*-factor *wR* and goodness of fit *S* are based on *F*^2^, conventional *R*-factors *R* are based on *F*, with *F* set to zero for negative *F*^2^. The threshold expression of *F*^2^ > σ(*F*^2^) is used only for calculating *R*-factors(gt) *etc*. and is not relevant to the choice of reflections for refinement. *R*-factors based on *F*^2^ are statistically about twice as large as those based on *F*, and *R*- factors based on ALL data will be even larger.

### Fractional atomic coordinates and isotropic or equivalent isotropic displacement parameters (Å^2^)

**Table:**

	*x*	*y*	*z*	*U*_iso_*/*U*_eq_	
O10	0.09705 (15)	−0.35189 (15)	0.55947 (8)	0.0309 (4)	
H10	0.0938	−0.3890	0.5168	0.046*	
O21	0.07886 (17)	−0.41878 (17)	0.40700 (9)	0.0371 (4)	
H21A	0.0106	−0.5029	0.4127	0.108 (13)*	
H21B	0.0326	−0.3487	0.3699	0.080 (10)*	
O1	0.15674 (17)	0.09639 (15)	0.59615 (7)	0.0299 (4)	
O14	0.0447 (2)	0.21621 (17)	0.69040 (8)	0.0424 (5)	
C16	0.2016 (2)	0.0987 (2)	0.42937 (12)	0.0332 (5)	
H16	0.1281	0.1288	0.4617	0.040*	
C5	0.2133 (2)	−0.0433 (2)	0.58766 (11)	0.0257 (5)	
C9	0.2434 (2)	−0.2746 (2)	0.66557 (11)	0.0283 (5)	
H9A	0.1778	−0.3283	0.6981	0.034*	
H9B	0.3403	−0.2774	0.6889	0.034*	
C11	0.3067 (2)	−0.4999 (2)	0.58931 (12)	0.0334 (5)	
H11A	0.2479	−0.5566	0.6239	0.040*	
H11B	0.2965	−0.5428	0.5388	0.040*	
C8	0.2462 (2)	−0.3443 (2)	0.58623 (11)	0.0267 (5)	
C2	0.1017 (3)	0.1045 (2)	0.66926 (11)	0.0319 (5)	
C12	0.4598 (3)	−0.5102 (2)	0.61430 (12)	0.0327 (5)	
C4	0.1930 (2)	−0.1211 (2)	0.65843 (10)	0.0260 (5)	
C6	0.2752 (2)	−0.0953 (2)	0.52416 (11)	0.0244 (5)	
C17	0.2193 (3)	0.1677 (3)	0.35963 (12)	0.0408 (6)	
H17	0.1571	0.2432	0.3454	0.049*	
C7	0.3324 (2)	−0.2498 (2)	0.53073 (11)	0.0281 (5)	
H7A	0.4338	−0.2469	0.5478	0.034*	
H7B	0.3288	−0.2945	0.4804	0.034*	
C15	0.2936 (2)	−0.0165 (2)	0.45162 (11)	0.0264 (5)	
C20	0.4029 (2)	−0.0591 (2)	0.40115 (11)	0.0325 (5)	
H20	0.4645	−0.1357	0.4143	0.039*	
C3	0.1265 (2)	−0.0318 (2)	0.70729 (11)	0.0308 (5)	
H3	0.1009	−0.0542	0.7572	0.037*	
C19	0.4203 (3)	0.0118 (3)	0.33181 (13)	0.0391 (6)	
H19	0.4938	−0.0171	0.2991	0.047*	
C18	0.3288 (3)	0.1249 (3)	0.31113 (12)	0.0412 (6)	
H18	0.3409	0.1723	0.2647	0.049*	
C13	0.5838 (3)	−0.5168 (3)	0.63321 (14)	0.0429 (6)	
H13	0.6818	−0.5221	0.6481	0.051*	

### Atomic displacement parameters (Å^2^)

**Table:**

	*U*^11^	*U*^22^	*U*^33^	*U*^12^	*U*^13^	*U*^23^
O10	0.0292 (9)	0.0298 (8)	0.0334 (8)	0.0001 (6)	−0.0032 (6)	−0.0002 (6)
O21	0.0357 (9)	0.0365 (9)	0.0391 (9)	−0.0021 (7)	0.0032 (7)	0.0071 (7)
O1	0.0433 (9)	0.0239 (8)	0.0228 (7)	0.0043 (7)	0.0059 (6)	−0.0005 (6)
O14	0.0641 (12)	0.0332 (10)	0.0302 (8)	0.0141 (8)	0.0060 (7)	−0.0034 (7)
C16	0.0351 (13)	0.0383 (13)	0.0263 (11)	0.0022 (10)	0.0027 (9)	0.0027 (9)
C5	0.0274 (11)	0.0224 (11)	0.0275 (10)	0.0011 (8)	0.0006 (8)	0.0005 (8)
C9	0.0311 (12)	0.0276 (11)	0.0262 (10)	0.0018 (9)	0.0017 (8)	0.0045 (9)
C11	0.0361 (13)	0.0273 (11)	0.0367 (12)	0.0042 (10)	0.0008 (9)	0.0006 (9)
C8	0.0245 (11)	0.0258 (11)	0.0296 (10)	0.0006 (9)	−0.0016 (8)	0.0017 (8)
C2	0.0389 (13)	0.0326 (12)	0.0243 (10)	0.0026 (10)	0.0024 (9)	−0.0039 (9)
C12	0.0388 (15)	0.0298 (12)	0.0296 (11)	0.0069 (10)	0.0045 (9)	−0.0020 (9)
C4	0.0270 (11)	0.0273 (11)	0.0238 (10)	−0.0027 (9)	−0.0016 (8)	0.0008 (8)
C6	0.0225 (10)	0.0273 (11)	0.0235 (10)	−0.0015 (8)	0.0013 (8)	−0.0014 (8)
C17	0.0456 (15)	0.0438 (14)	0.0328 (12)	0.0013 (12)	−0.0017 (10)	0.0099 (10)
C7	0.0296 (12)	0.0271 (11)	0.0278 (10)	0.0013 (9)	0.0025 (8)	−0.0019 (8)
C15	0.0279 (12)	0.0284 (11)	0.0227 (10)	−0.0049 (9)	−0.0007 (8)	−0.0016 (8)
C20	0.0339 (13)	0.0368 (13)	0.0269 (11)	−0.0016 (10)	0.0040 (9)	−0.0015 (9)
C3	0.0362 (13)	0.0343 (12)	0.0219 (10)	0.0019 (10)	0.0040 (9)	0.0011 (8)
C19	0.0399 (14)	0.0498 (15)	0.0281 (11)	−0.0069 (11)	0.0096 (9)	−0.0014 (10)
C18	0.0477 (15)	0.0494 (15)	0.0265 (11)	−0.0089 (12)	0.0006 (10)	0.0096 (10)
C13	0.0374 (15)	0.0505 (16)	0.0407 (13)	0.0106 (12)	−0.0011 (11)	−0.0062 (11)

### Geometric parameters (Å, °)

**Table:**

O10—C8	1.436 (2)	C11—H11B	0.9700
O10—H10	0.8200	C8—C7	1.533 (3)
O21—H21A	1.0020	C2—C3	1.437 (3)
O21—H21B	1.0021	C12—C13	1.179 (3)
O1—C2	1.384 (2)	C4—C3	1.341 (3)
O1—C5	1.397 (2)	C6—C15	1.473 (3)
O14—C2	1.215 (3)	C6—C7	1.521 (3)
C16—C17	1.386 (3)	C17—C18	1.383 (4)
C16—C15	1.405 (3)	C17—H17	0.9300
C16—H16	0.9300	C7—H7A	0.9700
C5—C6	1.344 (3)	C7—H7B	0.9700
C5—C4	1.444 (3)	C15—C20	1.403 (3)
C9—C4	1.492 (3)	C20—C19	1.387 (3)
C9—C8	1.528 (3)	C20—H20	0.9300
C9—H9A	0.9700	C3—H3	0.9300
C9—H9B	0.9700	C19—C18	1.381 (3)
C11—C12	1.463 (3)	C19—H19	0.9300
C11—C8	1.538 (3)	C18—H18	0.9300
C11—H11A	0.9700	C13—H13	0.9300
			
C8—O10—H10	109.5	C3—C4—C5	107.93 (18)
H21A—O21—H21B	107.9	C3—C4—C9	132.25 (18)
C2—O1—C5	106.88 (15)	C5—C4—C9	119.83 (17)
C17—C16—C15	120.6 (2)	C5—C6—C15	126.24 (19)
C17—C16—H16	119.7	C5—C6—C7	114.96 (17)
C15—C16—H16	119.7	C15—C6—C7	118.79 (16)
C6—C5—O1	125.43 (18)	C18—C17—C16	120.4 (2)
C6—C5—C4	126.35 (19)	C18—C17—H17	119.8
O1—C5—C4	108.21 (16)	C16—C17—H17	119.8
C4—C9—C8	109.49 (16)	C6—C7—C8	113.49 (16)
C4—C9—H9A	109.8	C6—C7—H7A	108.9
C8—C9—H9A	109.8	C8—C7—H7A	108.9
C4—C9—H9B	109.8	C6—C7—H7B	108.9
C8—C9—H9B	109.8	C8—C7—H7B	108.9
H9A—C9—H9B	108.2	H7A—C7—H7B	107.7
C12—C11—C8	114.41 (18)	C20—C15—C16	117.96 (19)
C12—C11—H11A	108.7	C20—C15—C6	119.86 (19)
C8—C11—H11A	108.7	C16—C15—C6	122.15 (19)
C12—C11—H11B	108.7	C19—C20—C15	120.8 (2)
C8—C11—H11B	108.7	C19—C20—H20	119.6
H11A—C11—H11B	107.6	C15—C20—H20	119.6
O10—C8—C9	106.40 (16)	C4—C3—C2	108.20 (18)
O10—C8—C7	108.67 (15)	C4—C3—H3	125.9
C9—C8—C7	110.64 (17)	C2—C3—H3	125.9
O10—C8—C11	107.82 (16)	C18—C19—C20	120.3 (2)
C9—C8—C11	111.90 (16)	C18—C19—H19	119.9
C7—C8—C11	111.21 (17)	C20—C19—H19	119.9
O14—C2—O1	119.52 (19)	C19—C18—C17	119.9 (2)
O14—C2—C3	131.7 (2)	C19—C18—H18	120.0
O1—C2—C3	108.78 (17)	C17—C18—H18	120.0
C13—C12—C11	178.7 (2)	C12—C13—H13	180.0
			
C2—O1—C5—C6	179.1 (2)	C5—C6—C7—C8	30.3 (3)
C2—O1—C5—C4	−0.7 (2)	C15—C6—C7—C8	−150.71 (18)
C4—C9—C8—O10	−66.2 (2)	O10—C8—C7—C6	60.3 (2)
C4—C9—C8—C7	51.7 (2)	C9—C8—C7—C6	−56.2 (2)
C4—C9—C8—C11	176.32 (17)	C11—C8—C7—C6	178.83 (16)
C12—C11—C8—O10	178.87 (16)	C17—C16—C15—C20	−0.2 (3)
C12—C11—C8—C9	−64.5 (2)	C17—C16—C15—C6	−178.1 (2)
C12—C11—C8—C7	59.8 (2)	C5—C6—C15—C20	156.6 (2)
C5—O1—C2—O14	−179.3 (2)	C7—C6—C15—C20	−22.3 (3)
C5—O1—C2—C3	0.9 (2)	C5—C6—C15—C16	−25.5 (3)
C6—C5—C4—C3	−179.6 (2)	C7—C6—C15—C16	155.6 (2)
O1—C5—C4—C3	0.2 (2)	C16—C15—C20—C19	0.7 (3)
C6—C5—C4—C9	0.0 (3)	C6—C15—C20—C19	178.67 (19)
O1—C5—C4—C9	179.82 (17)	C5—C4—C3—C2	0.3 (2)
C8—C9—C4—C3	154.0 (2)	C9—C4—C3—C2	−179.2 (2)
C8—C9—C4—C5	−25.5 (3)	O14—C2—C3—C4	179.5 (2)
O1—C5—C6—C15	−0.8 (3)	O1—C2—C3—C4	−0.8 (2)
C4—C5—C6—C15	179.0 (2)	C15—C20—C19—C18	−0.6 (3)
O1—C5—C6—C7	178.16 (18)	C20—C19—C18—C17	−0.1 (4)
C4—C5—C6—C7	−2.1 (3)	C16—C17—C18—C19	0.7 (4)
C15—C16—C17—C18	−0.5 (4)		

### Hydrogen-bond geometry (Å, °)

**Table:**

*D*—H···*A*	*D*—H	H···*A*	*D*···*A*	*D*—H···*A*
O10—H10···O21	0.82	1.94	2.735 (2)	163
O21—H21A···O10^i^	1.00	1.74	2.728 (2)	169
O21—H21B···O14^ii^	1.00	1.75	2.754 (2)	176
C13—H13···O21^iii^	0.93	2.47	3.236 (3)	139

Symmetry codes: (i) −*x*, −*y*−1, −*z*+1; (ii) −*x*, −*y*, −*z*+1; (iii) −*x*+1, −*y*−1, −*z*+1.
